# Editorial: Developmental trajectories in mental health between adolescence and adulthood

**DOI:** 10.3389/fpsyt.2023.1230996

**Published:** 2023-06-16

**Authors:** Marco Colizzi, Dario Marin, Antonella Trotta

**Affiliations:** ^1^Unit of Psychiatry, Department of Medicine (DAME), University of Udine, Udine, Italy; ^2^Department of Psychosis Studies, Institute of Psychiatry, Psychology and Neuroscience, King's College London, London, United Kingdom; ^3^Unit of Child and Adolescent Neuropsychiatry, Friuli Centrale Health University Authority, Udine, Italy; ^4^Department of Agricultural, Food, Environmental and Animal Sciences (DI4A), University of Udine, Udine, Italy; ^5^School of Health and Social Care, University of Essex, Colchester, United Kingdom; ^6^Social, Genetic and Developmental Psychiatry Centre, Institute of Psychiatry, Psychology and Neuroscience, King's College London, London, United Kingdom

**Keywords:** neurodevelopment, prevention, services, neurocognition, substance use

Several lines of research indicate that acquiring and applying a neurodevelopmental perspective to mental and behavioral problems can improve our ability to deliver earlier, more effective, and more personalized interventions (1, 2). Major points favoring such an approach are briefly summarized. Then, we present studies included in this Research Topic, which have tried to advance our knowledge neurodevelopmentally by tackling open questions in the field of psychiatry.

First, the conceptualization of psychiatric disorders as separated categories with a discrete etiological basis has been substantially superseded by the so-called “neurodevelopmental continuum” hypothesis. According to this hypothesis, an altered neurodevelopment would explain the partially overlapping phenotypes and nuanced clinical pictures observed in clinical practice. From this point of view, common features across multiple psychiatric disorders would reflect age-adjusted variations of common underlying dispositions, rather than distinctive forms of psychopathology (3, 4).

Second, a further refining of this framework integrates a gradient of severity into the neurodevelopmental hypothesis. In fact, the possible neurocognitive and psychopathological outcome would be more severe and have an earlier onset depending on the level of neurodevelopmental impairment (5). This is supported by epidemiological evidence that most mental disorders have their peak of incidence at different developmental time points but before young adulthood, with up to one in five people experiencing clinically relevant problems before the age of 25, 50% of whom by the age of 14 (6).

Third, inherent within the neurodevelopmental framework is the utility to avoid a photostatic representation of mental disorders in favor of a developmental trajectory. The brain maturation is characterized by systems that develop before or after others, differentially interact across development, and present with sensitive periods where they are particularly exposed to the disrupting effects of experiences. A neurodevelopmental perspective is thus needed to understand when and how to intervene to positively modify illness trajectories (2, 7).

Fourth, evidence for altered brain development, early onset, and different time courses across neuropsychiatric disorders highlights how important is to adopt a neurodevelopmental approach also to mental health prevention (1, 8). Studies indicate that the average delay between onset of symptoms and intervention is around 10 years, with school dropout affecting ~50% of students aged 14 and older who suffer from a psychiatric condition. It is thus not surprising that among people younger than 25 years old, mental health problems, especially anxiety and mood disorders, are the main cause of disability-adjusted life-years (DALYs), accounting for 45% of the global burden of disease, with problematic substance use being the main risk factor for incident DALY (9%) (9).

Fifth, research evidence supports the importance to reorient mental health services to the needs of the youth population (8). Mental healthcare has been traditionally oriented to offer support to adults and in acute settings (10). However, neuropsychiatric needs are often encountered in pediatric settings, with a potential increase following the COVID-19 pandemic (11, 12). It is worth mentioning that deinstitutionalization policies have not modified such a phenomenon, while recent years have seen the creation of new services for young individuals (8).

El Damaty et al. drawing from the Adolescent Development Study, propose a data-driven definition of neurocognitive age or maturity of adolescent cognitive development. Their model defines cognitive maturity as occurring along a continuum that develops from childhood into adolescence and emerging adulthood. Using latent factor estimates, the authors derive a Cognitive Maturity Index from behavioral performance on neurocognitive measures of inhibitory control, risky decision-making, and emotional processing, which have showed to be reliable predictors of cognitive skill development and life outcomes, such as IQ, risk for violence, and risk for substance use disorder, in a sample of children followed longitudinally from ages 11 to 18.

Adolescents' relationship with risk and sensitivity to reward is therefore crucial in the achievement of developmental milestones, to facilitate the transition to adulthood. However, the rapid changes in brain development and increased risk-taking might lead to negative outcomes, including substance use. The study of McQuaid et al. conducted with the same sample as El Damaty et al. uses cognitive and neuroimaging tasks to understand behavioral and neural risk markers that *precede* initiation of substance use in adolescence. The study shows neural differences in the prefrontal cortex activity as potential predictors to vulnerability to substance abuse. Despite showing similar risk-taking behavior, adolescents who use substances show in fact a different pattern of brain activity compared to non-users, specifically in the left anterior cingulate cortex and insula, known to be key neural regions involved in reward and risk-related decision.

Focusing on the interplay between cognitive and emotional development, Ren and Fishbein propose a prospective, longitudinal study, to investigate the neurodevelopmental consequences of marijuana use during adolescence. Their study highlights that early onset of marijuana use in adolescence is associated in the short term with exhibited deficits in verbal learning ability. Further, in late adolescence, a decline in emotion recognition ability into emerging adulthood is observed.

The period of young adulthood is characterized by specific developmental tasks, including separation from the families of origin and development of a sense of autonomy, defining one's own identity, building long-term social relationships, and establishing a professional career.

Adopting a systemic and developmental perspective on understanding disability, Levante et al. focus on the psychological dynamics involved in the relationship between siblings, particularly with reference to the phenomenon of “parentification”, when the siblings of a brother/sister with disabilities assume parent-like duties. The authors hypothesize that the siblings' distress and the quality of the relationship with parents would mediate the association between siblings' parentification and sibling relationship. A strength of this study is the focus on social support and perceived benefits of parentification as protective factors, and not only as risk factors. The paper in fact looks at the phenomenon of parentification through multiple lenses: it considers the positive effect of parentification on the functioning of siblings of people with disabilities, in terms of increased responsibility, empathy, and maturity, as well as its negative outcomes such as high levels of perceived responsibility and low quality of life, shame and concerns about the caregiver's role, and negative emotional adjustment. The study also explores the intergenerational dynamics between siblings of people with disabilities and their parents, as well as with their siblings with disabilities.

Studies included in this Research Topic highlight the complex neurodevelopmental picture that characterizes the years between adolescence and young adulthood. The combination of rapid brain development, cognitive skills, and emotional regulation functions, alongside the influence of the environment (including family and peers), makes these stages of life particularly challenging for young people. Articles in this Research Topic demonstrate the importance of identifying critical developmental windows to maximize the benefits of educational and clinical interventions, and the relevance of involving the family and peer system considering the age-specific needs of adolescent and young adults.

## Author contributions

All authors listed have made a substantial, direct, and intellectual contribution to the work and approved it for publication.
